# Howiesons Poort backed artifacts provide evidence for social connectivity across southern Africa during the Final Pleistocene

**DOI:** 10.1038/s41598-022-12677-5

**Published:** 2022-06-09

**Authors:** Amy M. Way, Paloma de la Peña, Eduardo de la Peña, Lyn Wadley

**Affiliations:** 1 Evolutionary Studies Institute, University of the Witwatersrand, PO Wits, Johannesburg, 2050 South Africa; 2 Geoscience and Archaeology, Australian Museum Research Institute, Australian Museum, Sydney, NSW 2010 Australia; 3 Department of Archaeology, School of Philosophical and Historical Inquiry, The University of Sydney, Sydney, NSW 2006 Australia; 4 McDonald Institute for Archaeological Research, Downing Street, Cambridge, CB2 3ER UK; 5 Center of Exploration of the Deep Human Journey, University of the Witwatersrand, PO Wits, Johannesburg, 2050 South Africa; 6 Department of Plants & Crops, Faculty of Bioscience Engineering, Ghent University, 9000 Ghent, Belgium; 7 Instituto de Hortofruticultura Subtropical y Mediterránea “La Mayora (IHSM‐UMA‐CSIC), Estación Experimental “La Mayora”, Málaga, Spain

**Keywords:** Archaeology, Social evolution, Evolution, Environmental social sciences

## Abstract

Examining why human populations used specific technologies in the Final Pleistocene is critical to understanding our evolutionary path. A key Final Pleistocene techno-tradition is the Howiesons Poort, which is marked by an increase in behavioral complexity and technological innovation. Central to this techno-tradition is the production of backed artifacts—small, sharp blades likely used as insets in composite tools. Although backed artifacts were manufactured for thousands of years before the Howiesons Poort, this period is marked by a phenomenal increase in their production. In this paper we test both social and environmental hypotheses to explain this phenomenon. We correlate environmental data with changing frequencies of backed artifact production at Sibudu and assess morphological similarity across seven sites in southern Africa. We find that these artifacts are made to a similar template across different regions and that their increased production correlates with multiple paleo-environmental proxies. When compared to an Australian outgroup, the backed artifacts from the seven southern African sites cluster within the larger shape space described by the Australian group. This leads us to argue that the observed standardized across southern Africa is related to cultural similarities and marks a strengthening of long-distance social ties during the MIS4.

## Introduction

**Figure 1:**
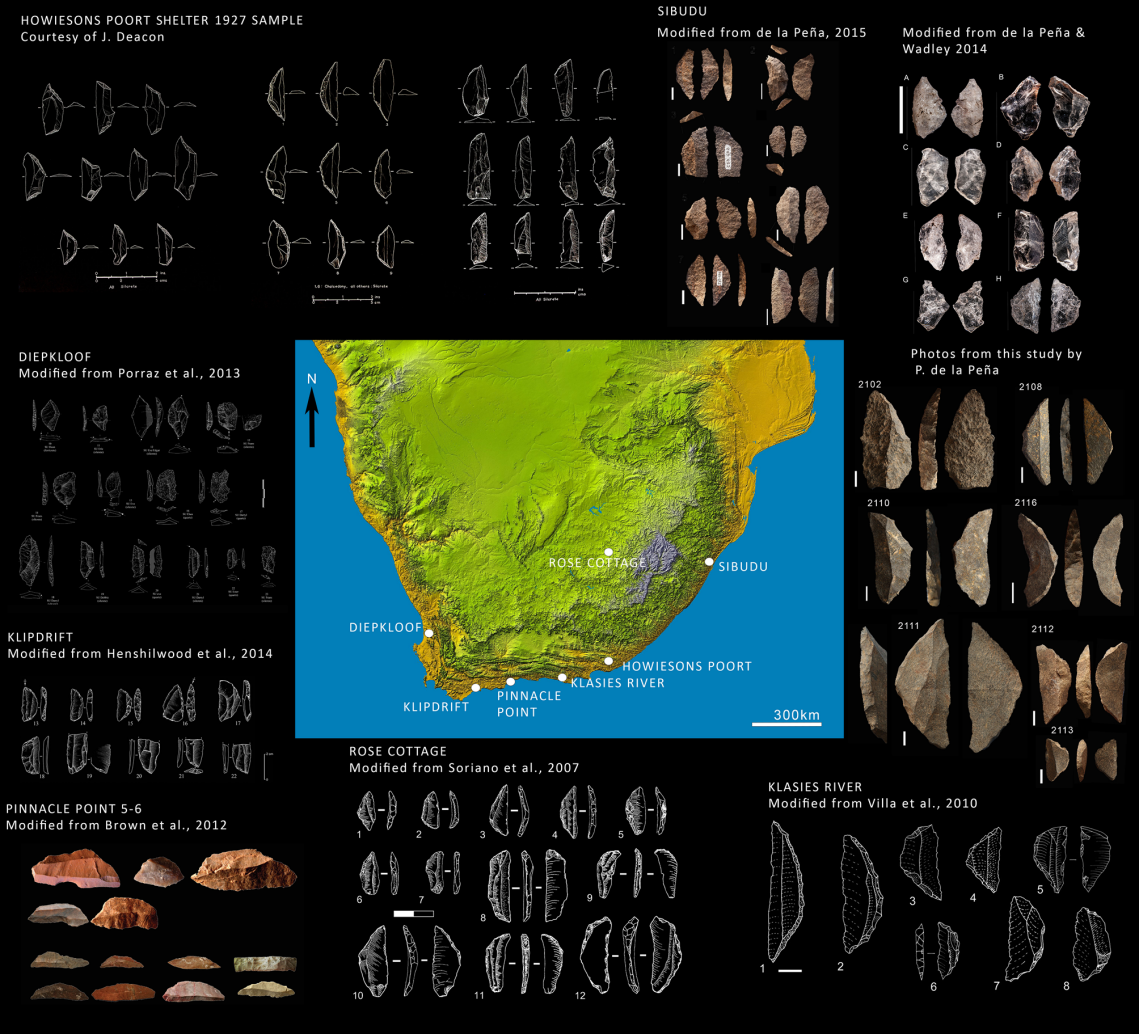
Map showing the location of the seven sites analyzed alongside a small selection of the backed pieces used for this analysis: Howiesons Poort Shelter eponymous site, Sibudu, Klasies River, Rose Cottage, Pinnacle Point 5–6, Klipdrift Shelter, and Diepkloof. The map is sourced from https://www.jpl.nasa.gov/images/srtm-data-release-for-africa-colored-height.The Howiesons Poort site artifacts are shown courtesy of J. Deacon^14^. The Sibudu crystal quartz (Grey Sand layer) are reproduced from^8^; dolerite (Grey rocky layer), to the left of the quartz are reproduced from^18^ and hornfels (Grey rocky layer), below the quartz, were photographed for this study. Klasies River backed pieces are from HP lower, middle and upper: (1,2,3,4,7 and 8) quartzite; (5,6,7) silcrete; modified from^64^. Rose Cottage opaline backed pieces; (1) Level EMD; (2) Level EMD; (3,5,6,7,8,12) Level MAS; (5,10) Level SUZ; modified from^38^. Pinnacle Point 5–6 backed pieces from the DBCS (HP) and SADBS layers, modified from^24^. Klipdrift Shelter’s backed pieces were modified from^65^ and come from layer PCA, PBC, PBA/PBB, layer PBD (quartz and silcrete). Diepkloof’s backed pieces are from the late HP on silcrete and quartz, drawings by M. Grenet, modified from^20^. This figure provides a visible representation of the variability of backed pieces in southern Africa. This figure was created with Adobe Photoshop CS6 www.adobe.com by PP.

The dramatic increase in backed artifact production during the Howiesons Poort in Southern Africa from 65 to 60 Ka^1^ has been variously attributed to either environmental or social shifts^2–7^. In this paper, we examine both social and environmental hypotheses. We present a morphological study of the backed artifacts of seven southern African Middle Stone Age sites to test whether similarities in shape are visible across great distances and different biomes. These assemblages are drawn from Sibudu, Klasies River Mouth, Pinnacle Point, Rose Cottage Cave, Howiesons Poort site, Klipdrift Shelter and Diepkloof (Fig. 1). To determine whether the results are the product of randomness or cultural similarity, the seven southern African sites are subsequently compared with an Australian outgroup. We also examine the evidence for changing climatic conditions during this time. We correlate the frequency of backed artifact discard with paleo-environmental proxies at Sibudu to examine whether increased backed artifact production can be explained as the consequence of deteriorating climatic conditions.

The Howiesons Poort technological tradition was first defined with large backed artifacts as a typological marker (^2^ and references therein). However, recently the Howiesons Poort’s backed artifacts have been related to the development of microlithism and complex solutions of hafting^8^. Moreover, backed artifacts in the southern Africa Middle Stone Age have served to demonstrate the widespread use of projectile technology^9^ and one of the oldest uses of barbs in prehistory^10^, which have been inferred as evidence of the first use of bow and arrow technology^11^. These developments have underpinned propositions regarding the advancement of logistic technological behavior during this period as a response to improved information sharing strategies following a period of increased scarcity and patchiness in resources, brought on by the beginning of MIS4^12,13^. In other cases, the production of these tools has been interpreted as providing evidence for developing social interchange^14,15^ and symbolic meaning^16^. Furthermore, there are different technologies and symbolic expressions (such as ostrich eggshell engravings, engraved ochre, hunting and logistical strategies^17–20^) associated with the Howiesons Poort which, for some, have been conceived as precocious, or exceptional^21^, and for others as manifestations of a broader Middle Stone Age development of behavioral complexity^22^.

While the Howiesons Poort is marked by the dramatic increase in production of backed artifacts, these artifacts were already in use in earlier periods within the Middle Stone Age^23^. At Sibudu Cave there is evidence of backed artifact manufacture as early as 77 ka^1^ and at Pinnacle Point at 71 ka^24^. At Diepkloof, Tribolo and colleagues^25^, have proposed a much earlier and longer duration for the Howiesons Poort. They suggest the Howiesons Poort lasted for closer to 50,000 years, starting approximately 105/109 ka and not vanishing until after 52 ka (see also the early date proposed for central Africa by^23^). As these dates do not follow established Still Bay to Howiesons Poort chronologies at key Still Bay sites such as Blombos, in this paper we follow the more widely held chronological framework proposed in Jacobs et al.^1^, which presents a shorter term chronology for the Still Bay and Howiesons Poort, coinciding with Marine Isotopic Stage 4. Furthermore, the short chronology aligns more closely with most of the dated sites for the Still Bay and Howiesons Poort. This chronology, like the longer chronology, shows that backed artifacts were known before the dramatic increase in production during the Howiesons Poort^23,24^. The question then becomes, why were these artifacts manufactured in such large numbers at this time if these tools were already known?

Archer^26^ observes that both site numbers and backed artifact discard rates increase during the Howiesons Poort across all of southern Africa and suggests that it is the interaction between increased climatic variability and increased population density which underpins the increased frequency in the production of this tool. Backed artefacts were found to correlate with proxy measures of demographic changes, while other tools, such as points, scrapers and denticulates did not show a statistically significant correlation^26^. Archer^26^ suggests that the resource pressure exerted by larger populations ‘triggered the use of certain complex technologies like backed artifact production systems’. This technological innovation allowed prey populations resilient to demographic pressure to be exploited more efficiently. Increased demographic density also facilitated the transmission of this technology through increased contact between neighboring groups. Other tools, such as points and scrapers, conversely, did not respond to demographic change in any convincing way in Archer’s model, possibly because they did not have the desired functions under these conditions or because the skills necessary to manufacture these artefacts were not transmitted by social structures influenced by increased demographic density^26^.

Backed artifacts, more than other tools, are seen as a multi-functional and multi-use tool which can optimize resource collection under unpredictable or fluctuating conditions^27^. Within the Howiesons Poort context in southern Africa, use-wear analyses have shown that backed artefacts were used for a wide range of activities, rather than being devoted to specific tasks^26^. While broad scale correlations have been made in Africa between the production of backed artifacts and climatic changes (^28^ and references therein), we test for the first time whether this correlation holds at the local level when high resolution paleoenvironmental proxies are compared with the frequency of backed artifact discard. We compare the timing of the appearance of backed artifacts and their frequency of discard at Sibudu with paleoclimatic proxies of different scales: local (the Sibudu Cave data from^29^) continental: marine core MD02-2594^30^, and global: the Vostok ice core, Antarctica^31^. We chose different types of paleoclimatic proxies to cover different areas and scales, and to assess whether agreement exists both internally between the different proxies as well as between the proxies and the archaeological data. Within the analyses we place emphasis on the high resolution paleoclimatic proxies from Sibudu Cave and compare these with the coarser-grained generalized datasets.

While assessing the strength of these correlations, we also note that these artifacts appear across different biomes in southern Africa^1^, and that southern Africa experiences much greater climatic diversity than the Northern Hemisphere^32^. This means that the appearance and abundance of these artifacts cannot be simply attributed to environmental conditions even if correlations are found. What the environmental data will establish is whether changing conditions provide a potential impetus for new social responses.

Alongside Cortell-Nicolau^33^, we assume that ‘geometric microliths reflect culturally significant morphometric variation’ which means that morphometric variation can be used ‘to understand continuity, discontinuity and patterns of evolutionary change’. This theory suggests that similarity in shape is related to social connectedness between multiple neighboring communities. This hypothesis sees the shared possession of a mutually recognizable tool as being both the consequence of increasing social ties over long distances and a signal of increasing inclusivity, facilitated through the shared possession of a mutually recognizable tool. If increasing social connectedness underpins the increased production of backed artifacts during the Late Pleistocene in Southern Africa, we would expect to see a wide distribution of similarly shaped artifacts across multiple biomes.

By examining the morphology of backed artifacts across southern Africa and correlating the frequency of discard with environmental proxies, we test whether the significant increase in backed artifacts during the Howiesons Poort can be explained as the consequence of social connectedness between multiple neighboring communities, and if so, whether these social ties can be seen as a response to increasing environmental instability^34,35^.

## Materials and methods

### Backed artifact data

We analyzed 459 complete backed artifacts from across seven sites in southern Africa (Fig. 1) to ask the question: can the backed artifacts from different Howiesons Poort sites be differentiated by shape? We photographed the complete backed artifacts from the Wadley Sibudu collection and compared these with published photographs and drawings of complete backed artifacts from Diepkloof^20^), Howiesons Poort Shelter^14^, Klasies River^36,37^, Klipdrift Shelter^19^, Rose Cottage Cave^38,39^ and Conard’s excavation at Sibudu^6^. The Pinnacle Point research team provided the .tps file for the Pinnacle Point backed artifacts from Brown et al.^24^. The Klipdrift Shelter research team provided drawings and images from their recent excavations. To test whether the shape patterns were meaningful, we subsequently added an outgroup from an unrelated place and time which the authors had access to. The outgroup consisted of 95 backed artefacts from a late-Holocene site in south-eastern Australia (Lake George). As this assemblage was from a different space–time location it could not be related in the same way that Howiesons Poort sites are related to one anotherThe number of artifacts from each site is shown in Table 1. Unfortunately, raw material data was not available for most of the specimens and so was not included in the analysis.

**Table 1:** The number of backed artifacts in this study by site, showing author and image reference.

Site	No	Author
Diepkloof	21	Porraz et al.^20^
Howiesons Poort	42	Deacon^14^
Klasies River	80	Singer and Wymer^36^
Klasies River	13	Wurz^37^
Klipdrift Shelter	21	Douze et al.^19^
Pinnacle Point	21	Brown et al.^24^
Rose Cottage	120	Harper^39^
Rose Cottage	12	Soriano et al.^38^
Sibudu	119	This paper
Sibudu	10	Will and Conard^6^
TOTAL (southern Africa)	459	
Lake George (Australia)	95	Way^52^ and (in prep.)
Total	554	

### GMM analysis

We employed 2D geometric morphometric (GMM) analysis as the most comprehensive method for describing the shape of an object from photographic data^40^. This method has proved effective in discriminating variability between backed artifacts in Later Stone Age Africa^41^. The methods developed by Brown et al.^24^ for examining the shape of backed artefacts in southern Africa were employed in the GMM analysis to facilitate comparative analyses in this region of the world. This involved placing 16 landmarks on the dorsal view of each artifact. Two of these landmarks were placed on the proximal and distal tips. These landmarks were considered ‘technologically homologous’ and were fixed. Seven of the remaining fourteen landmarks were placed along the backed edge and seven along the cutting edge. These fourteen landmarks were treated as sliding semi-landmarks. Rohlf’s^42^ programs TpsDig and TpsUtil were used to digitize the landmarks and create the .tps file. The .tps file is provided in the Supplementary Information (SI Dataset S1). The statistical analyses were then performed using R version 4.1.0^43^ and RStudio v.2.1, with Procrustes Superimpositions, Generalized Procrustes Analysis, Principal Component Analysis and Linear Discriminant Analysis performed and plotted with Momocs v. 1.3.2^44^. The RStudio and .csv files are provided in the Supplementary Information (SI Datasets S2 and S3).

Generalised Procrustes Analysis (GPA) served to translate, rotate and re-scale each object’s coordinates, without changing the relative distances of the landmarks to each other. This removed the absolute size of the objects and allowed the shapes of the different objects to be compared. This did not remove the allometry, or size-related changes, of the specimens. Allometry was retained as an essential source of variation in backed artefact specimens. The Principal Component Analysis (PCA) then examined proportional variance for each dimension in the study and plots were produced to visually compare the shape space of the assemblages. K-means clustering was used to obtain morphometric clusters, regardless of the site, so that we could assess the main shape clusters in the sample. A cross-validation analysis was conducted using Linear Discriminant Analysis results. The cross-validation table determined how accurately individual backed artifacts could be assigned to their site of origin. Together, these analyses of variance allowed the level of similarity in shape between the artifacts in the different assemblages to be quantified.

### Environmental data

The environmental analysis employed both global and local environmental data from Sibudu to reconstruct paleoclimatic conditions through theHowiesons Poort—Final Middle Stone Age (MSA) sequence. Local paleoclimatic proxies were constructed from Bruch et al.^29^, which utilized late Pleistocene fossil plant material from Sibudu to quantify climate and vegetation. The underlying Still Bay and Pre-Still Bay strata of Sibudu could not be included in our analysis, as the local environmental data did not extend to this depth. Global proxies were drawn from the Southern hemisphere climatic proxies developed from the Vostok Ice Core, Antarctica^31^, which describes global temperature shifts over the last 420,000 years and the Agulhas Core (MD02-2594) taken from marine sediment off the coast of Cape Town, which documents ocean temperature shifts over the last 80,000 years^30^. While it is somewhat contentious to use sea temperature data to assess archaeological shifts^32^, we have included it as it offers a proxy from a different area and scale and has adequate temporal depth for the analysis^45^. While emphasis was placed on the local proxies from Sibudu, including the Vostok and Agulhas proxies allowed us to also test our hypotheses at a broader scale. While the GMM analysis utilized only complete backed artifacts, the backed artifact assemblage for the environmental analysis included all backed artifacts from Sibudu (n = 513).

### Environmental analysis

Using these proxies, a data matrix was built by assigning the climatic proxies allocated to each archeological stratum. The archaeological strata were grouped as follows for the analysis: Howiesons Poort, Post-Howiesons Poort, Late Middle Stone Age and Final Middle Stone Age. The relationship between different climatic (i.e., temperature and precipitation) and environmental variables (i.e., leaf area index, greenness and vegetation cover) and the number of backed artifacts was assessed by means of a Spearman’s rank correlation matrix as these variables did not display a normal distribution. Leaf area index (LAI) is an important property of vegetation and equates to half of the total green leaf area per horizontal ground surface unit. LAI is one of the primary measures used to characterize the size and density of plant canopies. Global climatic types are mainly defined by the seasonality of annual temperatures and precipitation^46^. Vegetation types and biomes can therefore be considered “crystallized, visible climate”^47^. Accordingly, mean annual temperature and precipitation, together with proxies related to vegetation features were used to assess whether the number of backed artifacts corresponded with variation in environmental variables. By means of generalized linear models, we assessed whether a linear relationship between the environmental and (micro)climatic variables and the number of backed artifacts existed. As many of the archaeological strata at Sibudu do not contain any backed artifacts this resulted in a zero-inflated dependent variable. Accordingly, a zero-inflated Poisson error structure was used in all generalized linear models with a log link function. Other error structures were also tested (Poisson and quasi-Poisson), but Zero-Inflated Poisson rendered consistently lower AIC values. We did not utilize generalized linear models with more than one predictor variable because of the high degree of collinearity (e.g., among the different temperature variables, or among the precipitation variables). Therefore, the most representative predictors for different types of variables were tested separately against the number of backed artifacts. All statistical analyses and plots were carried out using the packages *ggcorrplot*, *ggcorrplot*, *msm*, *gapminder*, *tidyr, ggplot2, hmisc, and pscl for zero-inflated Poisson models, all* in R version 4.1.0^43^.

## Results

### GMM results

**Figure 2:**
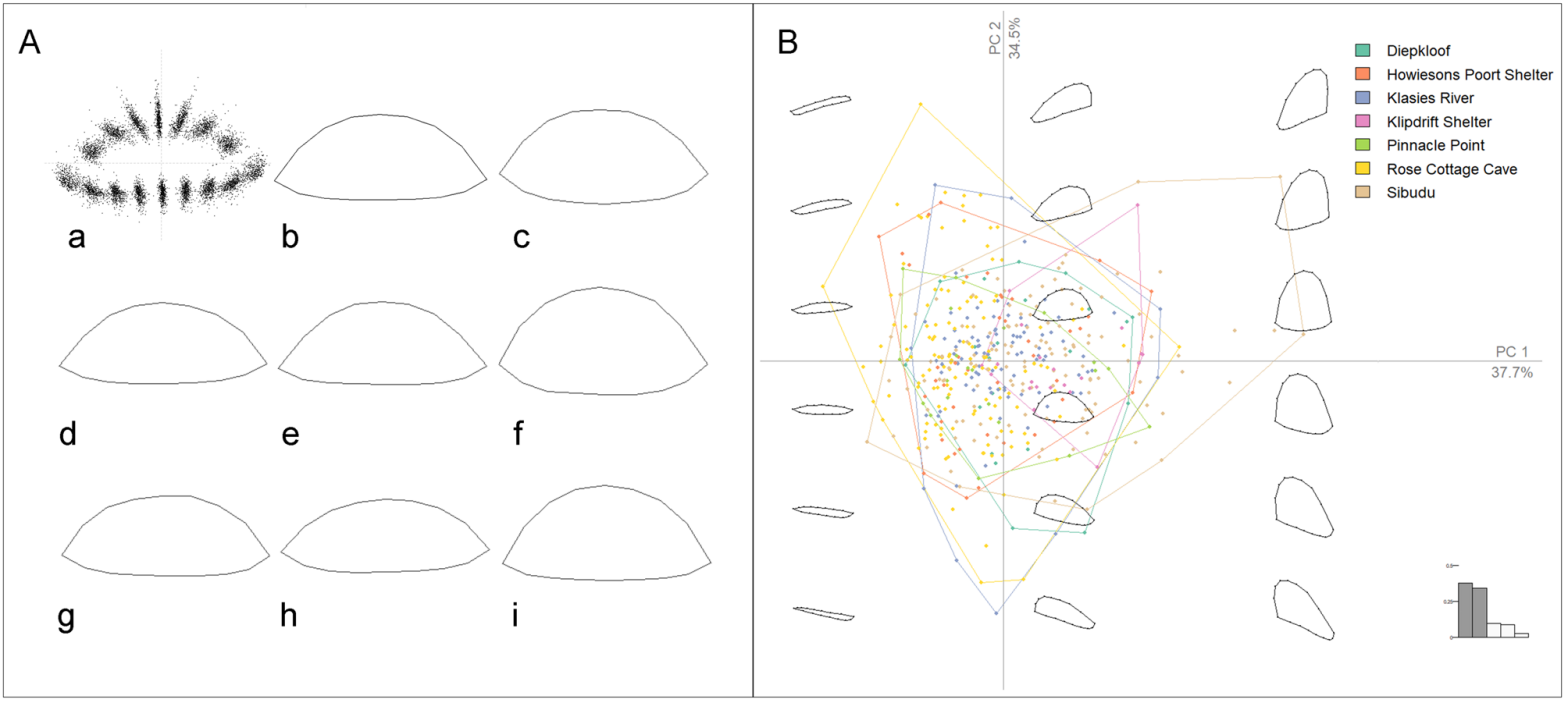
(**A**) GPA stack and mean shape of the backed artifacts from the seven southern African sites under study: (a) GPA stack of the 459 backed artifacts from the seven sites, (b) mean shape of all 459 southern Africa backed artifacts, followed by the mean shape of the backed artifacts from each site (c) Diepkloof, (d) Howiesons Poort Shelter, (e) Klasies River, (f) Klipdrift Shelter, (g) Pinnacle Point, (h) Rose Cottage Cave, (i) Sibudu; (**B**) Convex hulls representing variation along the first two principal components of the South African backed artifact assemblages. The percentage explained variance for each axis is in parentheses. Note the absence of clear between-group shape differences with the exception of a few outlying backed artifact shape configurations, principally from Sibudu. This figure was created in RStudio 1.4 www.rstudio.com by AW.

The results of the Generalised Procrustes Analysis (GPA) can be seen in Fig. 2A alongside the mean shape for the backed artifacts from each of the seven sites. While the mean shape remained similar across the sites, Sibudu, Diepkloof and Klipdrift Shelter had a mean shape which appeared to be wider with more obtuse tip angles than Howiesons Poort, Pinnacle Point, Klasies River Mouth and Rose Cottage Cave, which tended to be more elongated with more acute tip angles.

To assess shape variation across the seven sites a Principal Component Analysis (PCA) was performed on the landmark data and shape variations associated with individual PCs were obtained (see Fig. 2B). The first two Principal Component (PC) scores accounted for 72.2% of the total artifact shape variation. PC1 (37.7%) was related to artifact slenderness. For negative values, artifacts were narrowly elliptic-shaped with acute bases and tips; while for positive values, the artifacts presented rounder shapes with tip angles approaching 90°. PC2 (34.1%) represented convexity of the cutting margin along with skewing of the backed margin, with negative shape representations associated with convexity on the cutting margin and skewing to the left along the backed margin, and positive values associated with skewing to the right along the backed margin and concavity of the cutting margin. While the artifact shapes from the seven sites mostly overlapped with no clear site-level differentiation, the Sibudu assemblage, and to a lesser extent Rose Cottage Cave, had a range of shapes which extended beyond what was present at the other sites. The Sibudu assemblage had a small number of backed artifacts which were wider than those seen in the other six assemblages. To assess the main vectors defining the morphometry of the sample, we also investigated K-means clustering to obtain morphometric clusters, regardless of the site, however after assessing the optimal number of clusters, it was evident that the data consisted of a single cluster. The method for obtaining the optimal number of clusters is described in SI. This implies that while some differences in shape are present, the internal shape range was not sufficient to produce distinct clusters.

**Figure 3:**
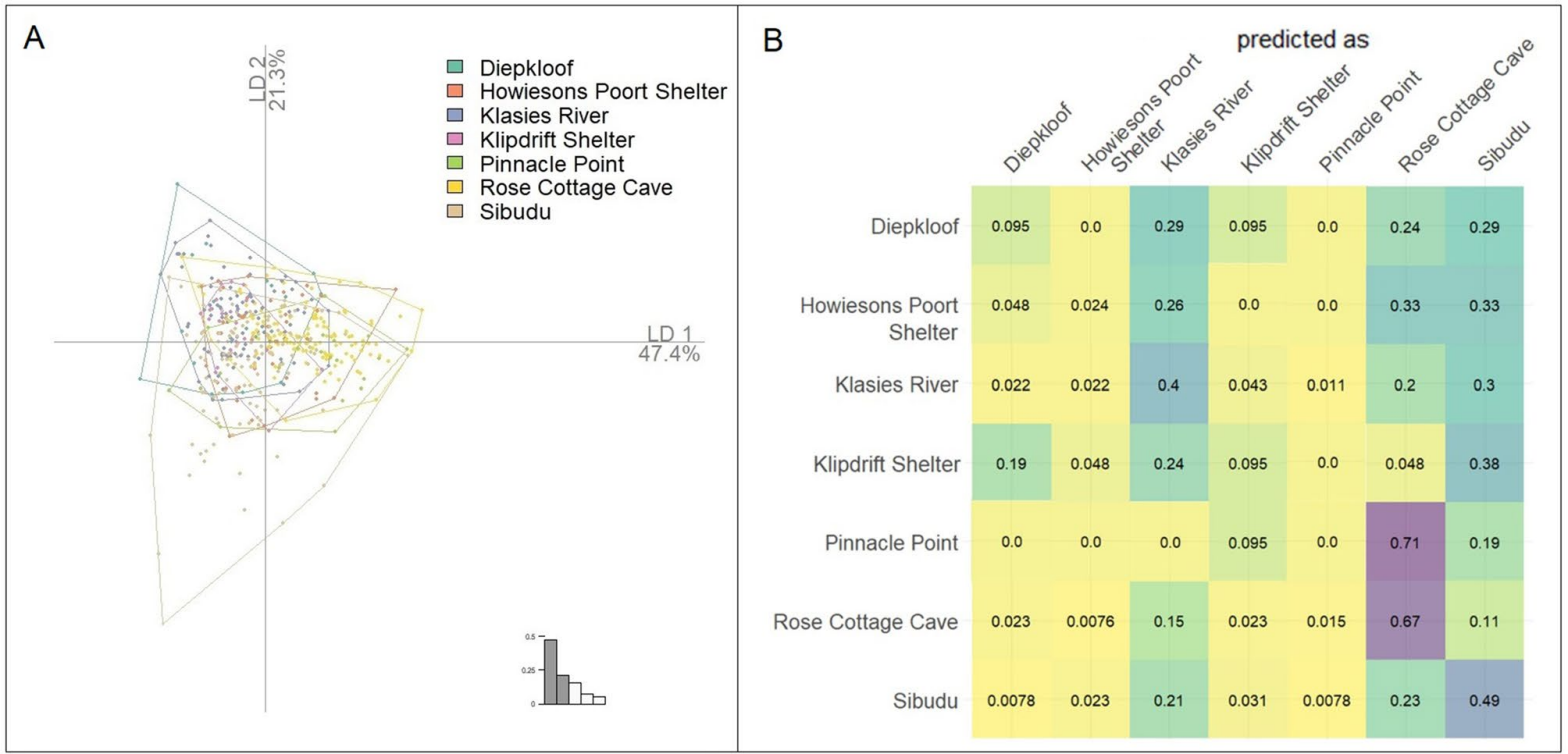
(**A**) Linear Discriminant Analysis (LDA) plot of the first two discriminants, which together account for 68.3% of between site shape variation. Note the lack of improvement in discrimination of sites, with the exception of a few outlying backed artifact shape configurations from Sibudu. (**B**) Results of cross validation tests over LDA inputs show the frequency the site of the backed artifact has been correctly predicted. The rows show the site and the columns show the prediction. Rose Cottage Cave had the highest frequency of correct prediction (67%) followed by Sibudu (49%), and Klasies River (40%). Diepkloof, Howiesons Poort Shelter, and Klipdrift Shelter were erroneously predicted in almost every iteration and Pinnacle Point was misclassified in every iteration. This figure was created in RStudio 1.4 www.rstudio.com by AW.

To further interrogate this pattern, the PCA was supplemented with a Linear Discriminant Analysis (LDA). LDA tests whether individual artifacts can be assigned to pre-defined groups. The LDA was based on all 31 PCs, which together explained 100% of total shape variation. The results are plotted in Fig. 3A. As with the PCA, there was little discrimination between the sites, with the exception of a few outliers from Sibudu.

To further test the robustness of these results a cross validation test over LDA inputs was conducted. The results show how many times the site of each backed artifact was correctly predicted. As can be seen in Fig. 3B little success was seen across the groups in terms of predicted membership, with the highest level of prediction (67%) for Rose Cottage Cave. Diepkloof, Howiesons Poort and Pinnacle Point were mis-classified in almost every iteration. When cross validation classification accuracy is high, the data support the occurrence of shape differences between groups. However, when the percentage of correctly classified specimens is low, as we see here, there is an absence of support for the occurrence of shape differences between groups.

**Figure 4:**
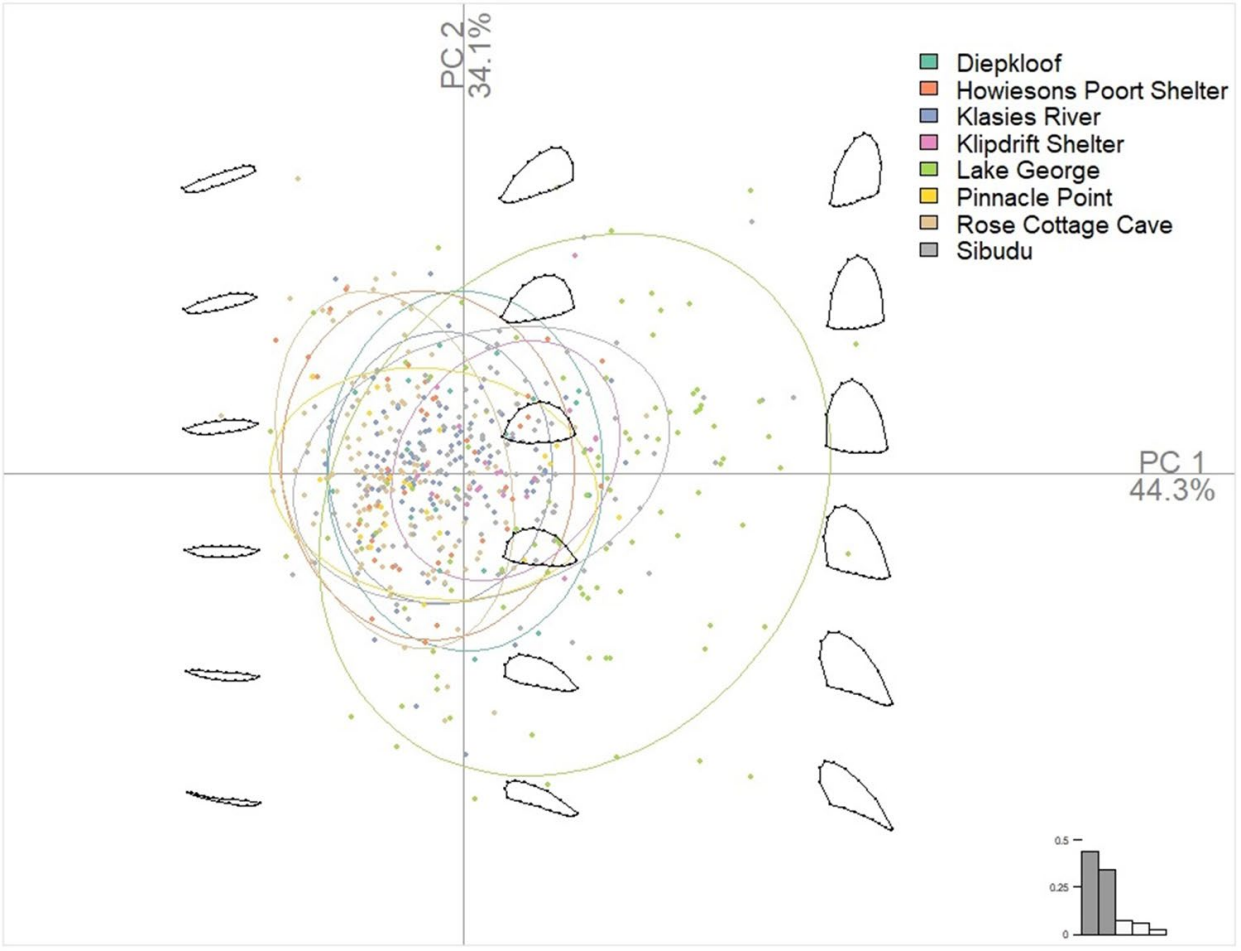
Scatter plot of PC1 and PC1 for the seven southern African and one Australian (Lake George) backed artefact samples with confidence ellipses drawn at the 95% probability level and PCA morphospace shown. This figure was created in RStudio 1.4 www.rstudio.com by AW.

To assess whether this homogeneity was related to cultural similarities, rather than a random component, we compared the seven southern African sites (n = 459) with an outgroup from an unrelated place and time. The outgroup consisted of the backed artefacts from a single late-Holocene site in south-eastern Australia, Lake George (n = 95). The results are plotted in Fig. 4. As can be seen, the southern Africa artefacts cluster within a limited area of the larger shape-space described by the smaller Australian sample. This means that only a subset of the backed artefact shapes manufactured within the Australian context were made across southern Africa. This supports the proposition that within southern African these artefacts were consistently made to a similar template, selected from a larger possibility of potential shapes.

### Environmental results

**Figure 5:**
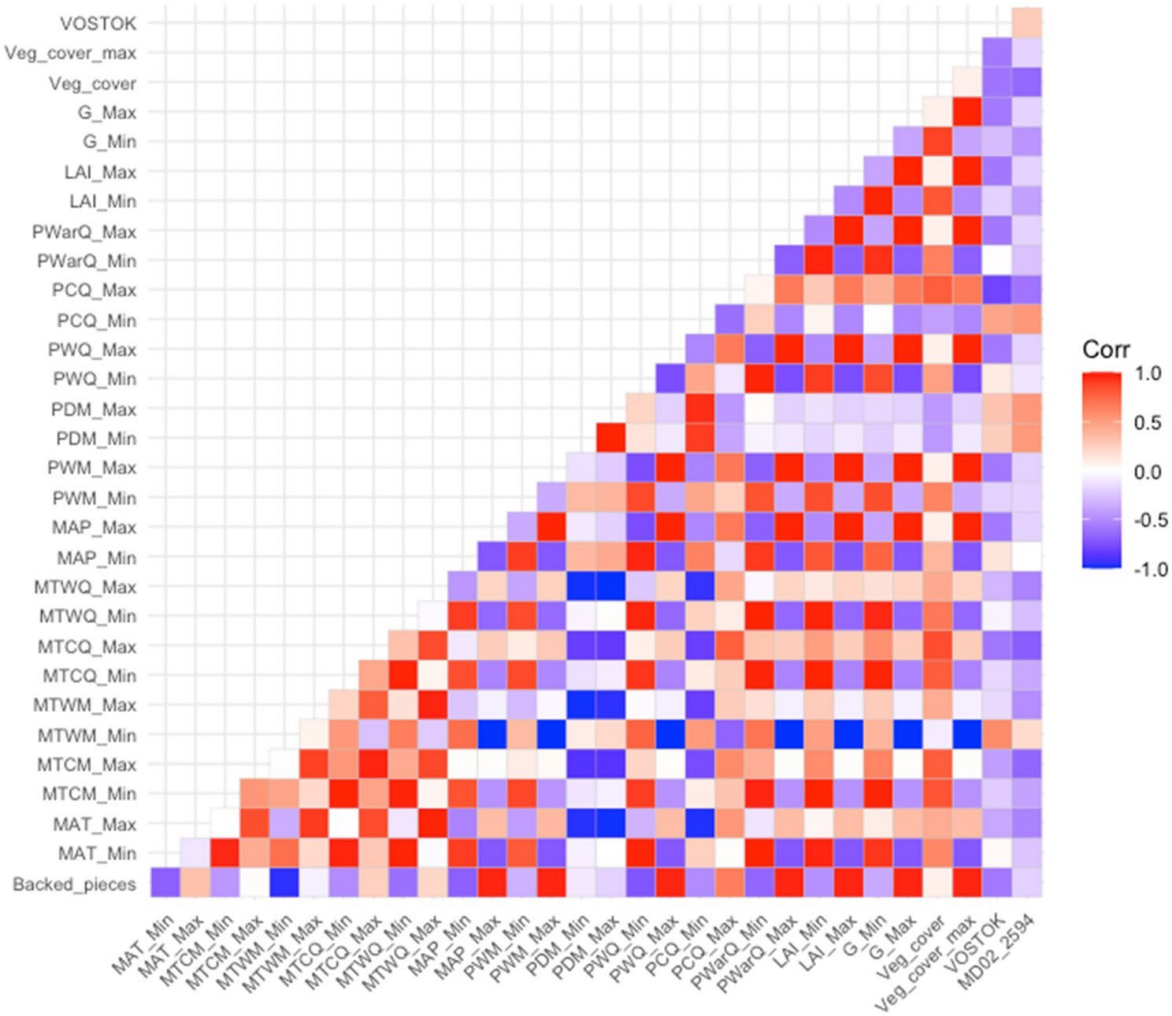
Correlation matrix between environmental, climatic variables and the number of backed artifacts. Color squares indicate significant Spearman rank correlation coefficients (p ≤ 0.05), white squares indicate non-significant correlation coefficients. Where BP stands for backed pieces, *MAT_Min* mean annual temperature (minimum), *MAT_Max* mean annual temperature (maximum), *MTCMin* minimum temperature of the coldest month (minimum), *MTCM_Max* minimum temperature of the coldest month(maximum), *MTWM_Min* maximum temperature of the warmest month (minimum), *MTWM_Max* maximum temperature of the warmest month (Maximum), *MTCQ_Min* mean temperature of the coldest quarter (Minimum), *MTCQ_Max* mean temperature of the coldest quarter (maximum), *MTWQ_Min* mean temperature of the warmest quarter (minimum), *MTWQ_Max* mean temperature of the warmest quarter (maximum), *MAP_Min* mean annual precipitation (minimum), *MAP_Max* mean annual precipitation (maximum), *PWM_Min* precipitation of the wettest month (minimum), *PWM_Max* precipitation of the wettest month (Maximum), *PDM_Min* precipitation of the driest month (minimum), *PDM_Max* precipitation of the driest month (maximum), *PDQ_Max* precipitation of the driest quarter (maximum), *PWQ_Max* precipitation of the wettest quarter (maximum), *PCQ_Min* precipitation of the coldest quarter (minimum), *PCQ_Max* precipitation of the coldest quarter (maximum), *PWQ_Min* precipitation of the warmest quarter (minimum), *PWQ_Max* precipitation of the warmest quarter (maximum), *LAI_Min* leaf area index (minimum), *LAI_Max* leaf area index (maximum), *G_Min* greenness (minimum), *G_Max* greenness (maximum), *Veg_Cover* vegetation cover (minimum), *Veg_cover_max* vegetation cover (maximum), *VOSTOK* Vostok temperatures, *MD022594* Temperatures from MD02-2594. Number of backed artifacts = 513. This figure was created in RStudio 1.4 www.rstudio.com by EP.

**Figure 6:**
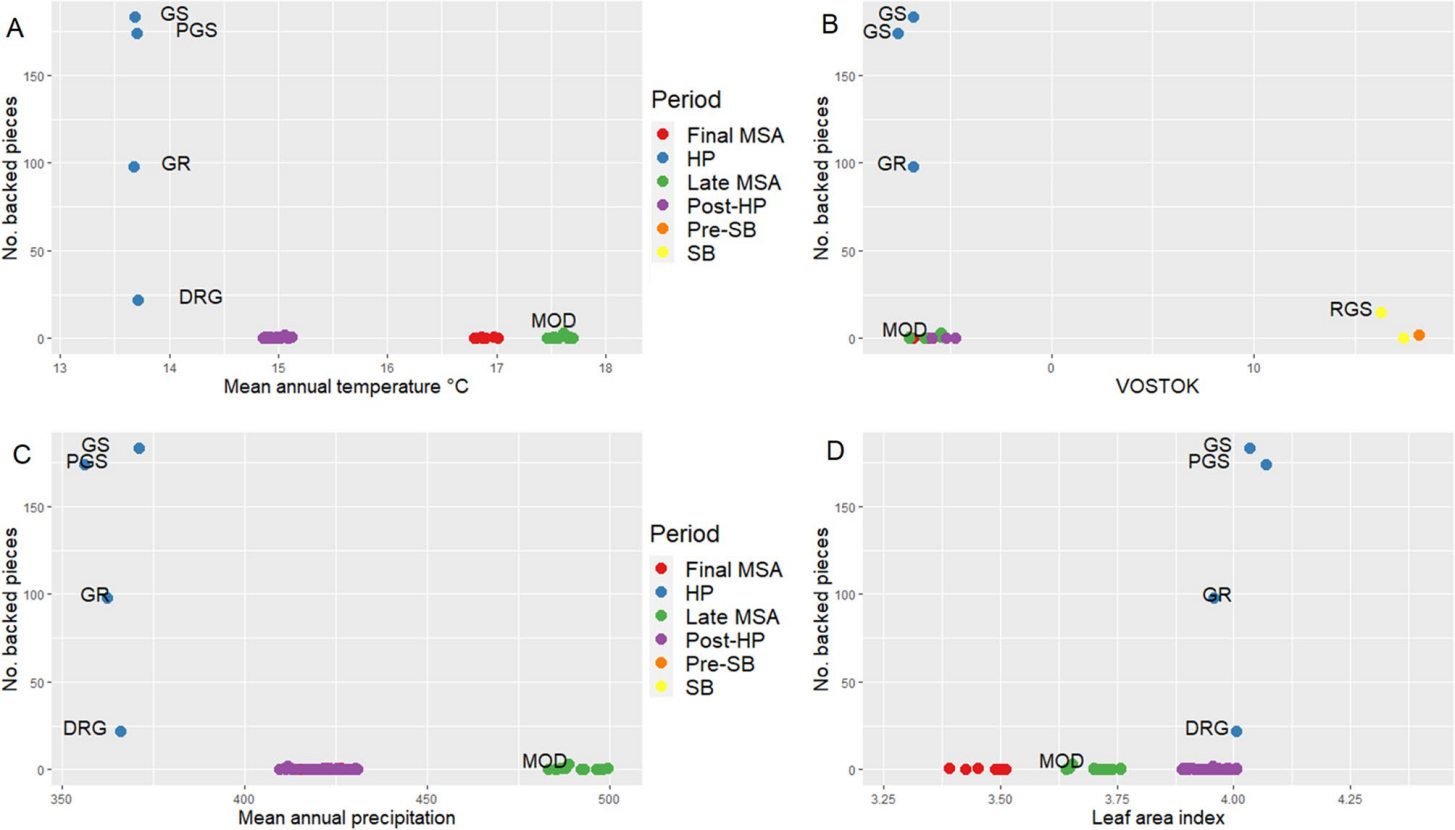
Scatter dot plots comparing the number of backed artifacts by technological group with (**A**) Mean annual temperature (minimum) (Sibudu). (**B**) Vostok global temperatures. (**C**) Mean Annual Precipitation (minimum) (Sibudu). (**D**) Leaf area index (Sibudu). Data for Sibudu from^29^. Data from MD02-2094 from^30^. This figure was created in RStudio 1.4 www.rstudio.com by EP.

Spearman rank correlations yield correlation coefficients that range from -1 to + 1. For each coefficient a p-value is calculated, and it indicates the probability of obtaining the observed pivot (or a more extreme one) if the null hypothesis is true. The number of backed pieces was mainly correlated with temperature proxies (i.e., Mean annual temperature (Minimum), Mean annual temperature (Maximum), Maximum temperature of the warmest month (Minimum), Mean temperature of the warmest quarter (Minimum), Vostok temperatures) and precipitation proxies (i.e., Mean annual precipitation (Minimum), mean annual precipitation (Maximum), Precipitation of the wettest month (Maximum), Precipitation of the driest quarter (Maximum), Precipitation of the wettest quarter (Maximum), Precipitation of the coldest quarter (Minimum), Precipitation of the coldest quarter (Maximum), Precipitation of the warmest quarter (Maximum)) and Leaf area index (Maximum) (Table S1). The correlation plot among climatic and environmental proxies pointed to a high degree of collinearity (i.e., correlation among used variables) (Fig. 5).The correlation analysis highlights the variation in the frequency of backed artifacts at Sibudu as a function of several paleoenvironmental proxies (Table 2, Fig. 6, Table S1).

**Table 2:** Statistic GLMs (with zero-inflated poisson error structures and a log link function) at the number of backed pieces and different environmental/climatic proxies at local (i.e. Mean Annual Temperature (Minimum) = MAT, Mean Annual Temperature (Max) = MATM, Mean Annual Temperature Warmest Month = MTWM_Max, Mean Annual Precipitation (Minimum) = MAP_Min, Precipitation Wetest Month (Minimum) = PWM_M, Precipitation Driest Month (Minimum) = PDM_Min, Leaf Area Index (Minimum) = LAI_Min, Greenness (Minimum) = G_Min) and global level (MD02_25_94 and VOSTOK).

	Estimate	Std. error	Z value	Pr(|z|)
**Temperature**				
MAT	− 2.40	0.13	− 17.88	< 0.01
MATM	0.97	0.04	19.41	< 0.01
MTWM_Max	0.06	0.03	1.78	0.07
**Precipitation**				
MAP_Min	− 0.007	0.004	− 17.59	< 0.01
PWM_M	− 0.21	0.02	− 8.82	< 0.01
PDM_Min	− 0.37	0.03	− 10.3	< 0.01
**Vegetation**				
LAI_Min	− 29.45	3.10	− 9.78	< 0.01
G_Min	− 7.02	0.64	− 10.85	< 0.01
Vegetation cover	0.17	0.03	4.61	< 0.01
**Global temperature proxies**				
MD02_2594	− 0.58	0.11	− 4.92	< 0.01
VOSTOK	− 0.12	0.01	− 8.34	< 0.01

Where bold indicate those proxies with a p ≤ 0.05.

In particular, higher frequencies of backed artifacts (as documented in the strata for Howiesons Poort) are related with shifts in the annual range of temperatures, i.e., the difference between the hottest and the coldest months by taking monthly mean temperatures, as shown by the negative and positive correlation with the minimum and maximum annual temperatures respectively. Similarly, variations in the different precipitation proxies are also related with higher frequencies in the number of backed artifacts. In general, a negative correlation between precipitation and the frequency of backed artifacts is observed on an annual basis but also for different seasonal proxies (i.e., precipitation of the driest quarter, precipitation of the warmest quarter, precipitation of the coldest quarter). The lack of unequivocal correlations for the different temperature and precipitation, or for contrasting correlation indexes for the minimum and the maximum values of related proxies, underpins important shifts in the seasonal ranges. Changes in annual and seasonal temperature and precipitation ranges are directly linked with shifts in the composition and abundance of the vegetation layer as acknowledged by Bruch et al.^29^. During the period of prolific backed artifact production, the Howiesons Poort, there was a local change towards cooler and less humid conditions. In addition, this period also saw the highest variation (i.e. amplitude range between maximum and minimum values) for leaf area index, greenness and vegetation cover.

Similarly, the zero-inflated Poisson regression models (GLM) (Table 2) showed a significant or nearly significant (p ≤ 0.10) response of the number of backed artefacts to mean annual temperatures, Vostok global annual temperature and the leaf area index. The relationship between the number of backed artifacts and other proxies was also investigated. It was consistently shown (except for sea surface temperatures from MD02-2594) that the high number of backed artifacts during the Howiesons Poort clustered apart from the backed artifacts from other archeological periods.

## Discussion

The key question asked in this paper is whether the increase in production of backed artifacts during the Howiesons Poort is the product of a social response to changing environmental conditions. To test this, we examined its two implications. Firstly, whether the nature of backed artifacts, as measured through their morphological similarity, points to an increase in social networking, and secondly, whether there was a correlation with the incidence of backed artifacts and climatic conditions. Our analyses show a correlation between backed artifact discard and environmental shifts towards drier and colder conditions as well as high levels of similarity in backed artifact shape across southern Africa.

We suggest increased cross-group connectivity underpins the high level of morphological similarity observed across this large geographical area. Multiple authors have argued for an increase in social coalescence across southern Africa during the Howiesons Poort (^48,49^ and references therein). The impressive technological and symbolic innovations associated with the Howiesons Poort (including the backed artifacts) are seen to relate to new patterns of connectivity, visible in changes in mobility, settlement systems, and foraging strategies^12,13,50^. Our analysis supports this hypothesis, with the observed similarity in backed artifact shape providing strong support for long distance social ties. Early parallel expressions of backed artifacts across multiple sites in southern Africa hint at the possibility that these patterns of connectivity began in an earlier period^48^.

In addition to being seen as the material consequence of increased connectivity, backed artifacts could also have reinforced social ties. The shared possession of ‘a mutually recognizable morphotype’ could have served to signal a level of shared cultural affiliation^34^. Previous hypotheses regarding backed artifacts in southern Africa have stressed the importance of their symbolic role^16^. These models have principally centered on exchange. They propose, through analogy with social interchange systems such as the hxaro gift-giving partnership of the Kalahari San, as described by Wiessner^51^, that backed artifacts functioned symbolically through exchange^14^.

While we suspect that raw material properties are irrelevant when secondary shaping takes place, due to the level of morphological agreement seen across the southern African assemblage, we were not able to investigate the role of raw material as this data was not available for many of the specimens under study. At Sibudu, most of the backed artifacts were made on local material^6,52^, which leads us to conclude that the increase in backed artifact manufacture was principally a product of increased knowledge sharing and copying, rather than direct trade or gift-giving. As backed artifacts regularly form part of complex tools, teaching and emulating must have involved not only the blank production and retouch methods required to produce the backed artifact itself, but also the hafting strategies and glue recipes needed to create the final multifaceted tool^10,53^. This implies complex mechanisms of transmission^48^.

We found a correlation between several environmental proxies and the production of backed artifacts at a high-resolution scale at Sibudu, and broadly across southern Africa. A correlation at Sibudu was observed between the frequency of backed artifact discard and a decrease in precipitation, temperature, leaf area index, and global temperatures. This accords with previously observed correlations between the Howiesons Poort and the beginning of MIS 4, which is characterized by cooler and less humid conditions in southern Africa^48,50,54–56^; but see^57^ and^58^.

It has been argued that environmental stress stimulated the production of backed artifacts^12,13,15,59–62^. The environmental argument, which tends to follow the risk hypothesis^27^ stresses a correlation between instability in hunting and foraging conditions and the manufacture of backed artifacts as a multi-functional and multi-use tool, which facilitates exploitation in uncertain or unstable environmental conditions^12,13,63^. Recently, however, Archer^28^ has correlated an *improvement* in the habitability of an area, or the ‘carrying capacity’ (essentially the amount of carbon-based edible biomass) with the production of backed artifacts under a hypothesis which sees backed artifacts appearing in great numbers during periods of population increase.

As we observed sustained morphological similarity across a distance of more than 1200 km and different ecological niches, we do not see the proliferation in backed artifacts as a functional response to changing environmental conditions. Rather, the increase in production should be seen as part of a socially mediated response to those changes, with strengthening social ties facilitating access to scarce, perhaps unpredictable resources. Importantly, this aligns with Archer’s^26^ central hypothesis that it is increased demographic pressure which may have both triggered the use of this complex technology and facilitated its transmission.

## Conclusion

During the Howiesons Poort backed artifacts are produced in enormous numbers across southern Africa, and it is this abundance which speaks to their success in this region at this time. Our morphometric analysis demonstrates that the Howiesons Poort backed artifacts are made to a similar template across great distances and multiple biomes. At Sibudu, the increase in frequency of backed artifact discard correlates with high-resolution paleo-environmental proxies. Together, these analyses provide new insight into the strength of social ties across southern Africa during the Howiesons Poort, and suggest that it was the strength of this social network which allowed populations to prosper in the face of changing climatic conditions. These findings hold global implications for understanding how expanding social networks contributed to the expansion of modern humans out of Africa and into new environments across Eurasia.

## Supplementary Information

Supplementary Information.
