# Supplementary material for: Howiesons Poort backed artifacts provide evidence for social connectivity across southern Africa during the Final Pleistocene
Source: Sci Rep. 2022 Jun 9;12:9227. doi: 10.1038/s41598-022-12677-5 (PMC9184481; doi:10.1038/s41598-022-12677-5)
Supplement: Supplementary file 1 — Supplementary Information. [file 41598_2022_12677_MOESM1_ESM.docx]

**Supplementary Information for**

Howiesons Poort backed artifacts provide evidence for social connectivity across southern Africa during the Final Pleistocene

This file includes:

Supplementary text

Fig S1

Table S1

Legends for Dataset S1 to S7

Other supplementary materials for this manuscript include the following:

Datasets S1 to S7

Supporting Information Text

**Change through time**

At Pinnacle Point, Brown et al. (1) argue for continuity in backed artefact production from the first expression around 71ka through to the peak period of production 61ka. Similarly Sibudu and Klasies River backed artefacts first appear in the Pre-Still Bay and Still Bay layers, respectively (2-4). These early parallel expressions suggest that the social networks relied upon during the Howiesons Poort had their roots in an earlier period. The broad similarities noted within Still Bay Point shapes supports the notion that widespread coordination was established before the Howiesons Poort (5-7). Further studies are needed to assess morphological change in backed artefacts through time and the potential long-term perseverance of shared norms across southern Africa.

**Method for determining optimal number of clusters**

The optimal number of clusters was assessed using all PCs and only the first 13 PCs (which accounted for 99% of the variation). The elbow method did not return a clear flexion point, indicating no clear point to differentiate clusters. To check the validity of this, the gapstat method was also used. The gapstat method returned 1 cluster for both datasets, aligning with the results of the elbow method (see Fig. S1). This indicates that clusters are not present in the data. The code for determining the optimal number of clusters is provided in **SI Dataset S3.**

**
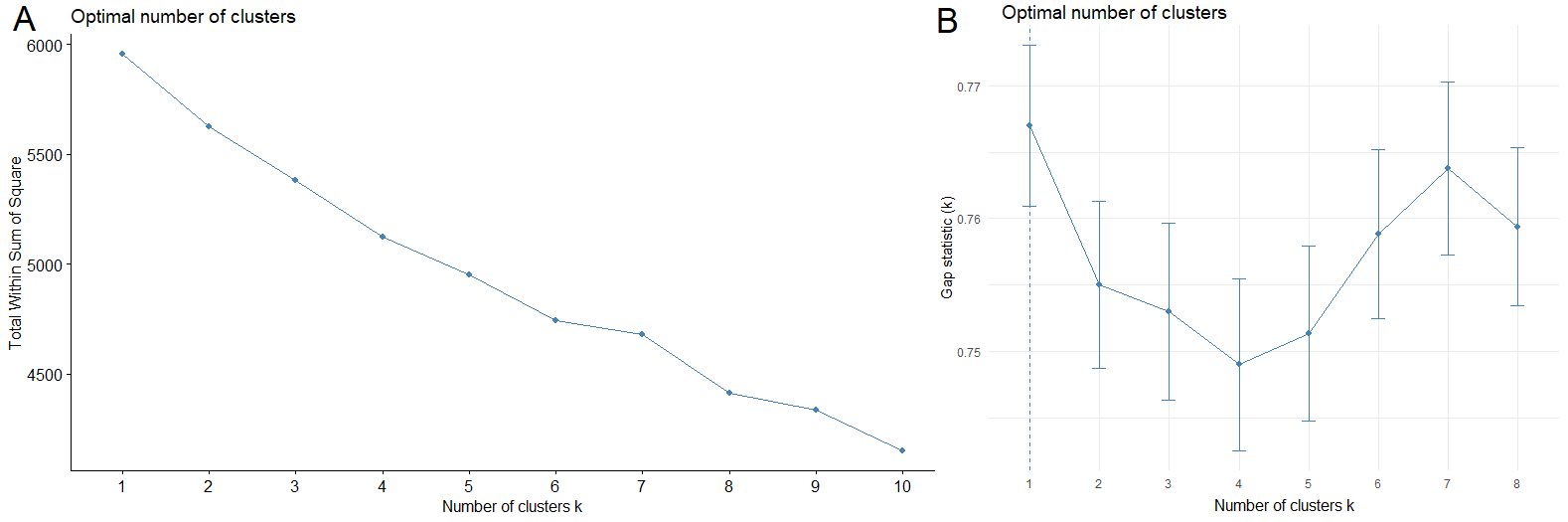
**

**FIG S1** Plots to determine the optimal number of clusters (dataset = PCs 1-3, which account for 99% of the variation). A. the elbow method, B. the gapstat method.

**Table S 1 Spearman rank coefficients for the correlation between the number of backed artefacts and various environmental/climatic proxies at local and global level. Where * p ≤ 0.05, ** p ≤ 0.01, *** p ≤ 0.005**

| Local climatic/environmental proxies | No. backed artifacts |
| --- | --- |
| Mean annual temperature (Minimum) | **-0.40**** |
| Mean annual temperature (Maximum) | **0.29*** |
| Minimum temperature of the coldest month (Minimum) | -0.18 |
| Minimum temperature of the coldest month(Maximum) | 0.10 |
| Maximum temperature of the warmest month (Minimum) | **-0.86***** |
| Maximum temperature of the warmest month (Maximum) | 0.02 |
| Mean temperature of the coldest quarter (Minimum) | -0.22 |
| Mean temperature of the coldest quarter (Maximum) | 0.24 |
| Mean temperature of the warmest quarter (Minimum) | **-0.33*** |
| Mean temperature of the warmest quarter (Maximum) | 0.20 |
| Mean annual precipitation (Minimum) | **-0.46**** |
| Mean annual precipitation (Maximum) | **0.86***** |
| Precipitation of the wettest month (Min) | -0.14 |
| Precipitation of the wettest month (Maximum) | **0.86***** |
| Precipitation of the driest month (Minimum) | -0.13 |
| Precipitation of the driest month (Maximum) | -0.19 |
| Precipitation of the driest quarter (Maximum) | **-0.47***** |
| Precipitation of the wettest quarter (Maximum) | **0.86***** |
| Precipitation of the coldest quarter (Minimum) | **-0.41**** |
| Precipitation of the coldest quarter (Maximum) | **0.48***** |
| Precipitation of the warmest quarter (Minimum) | **-0.36***** |
| Precipitation of the warmest quarter (Maximum) | **0.86***** |
| Leaf area index (Minimum) | -0.20 |
| Leaf area index (Maximum) | **0.86***** |
| Greeness (Minimum) | -0.12 |
| Greenness (Maximum) | **0.86***** |
| Vegetation cover (Minimum) | 0.14 |
| Vegetation cover (Maximum) | **0.86***** |
| Global climatic proxies | |
| VOSTOK-Temperatures | **-0.57*** |
| Temperatures from MD02-2594 | -0.20 |

***The following files are available at:***

[***https://gitfront.io/r/user-8560564/04ce84028f33471cd870d86c50f8e6fb0b53aacc/Howiesons-Poort-backed-artifacts/***](https://gitfront.io/r/user-8560564/04ce84028f33471cd870d86c50f8e6fb0b53aacc/Howiesons-Poort-backed-artifacts/)

**SI Dataset S1 (459_SA_BA.tps)**

The .tps file of the landmark data for the 459 southern African complete backed artifacts in the GMM analysis.

**SI Dataset S2 (459_SA_BA_place.csv)**

The .csv file containing site information for the 459 southern African complete backed artifacts in the GMM analysis.

**SI Dataset S3 (554_SA_AUS_BA.R)**

The R file containing the code used to complete the GMM analysis for both the southern African analyses and the additional analysis with the Australian outgroup.

**SI Dataset S4 (554_SA_AUS_BA.tps)**

The .tps file of the landmark data for the 459 complete backed artifacts in the GMM analysis from southern Africa in addition to the 95 complete backed artifacts from Australia.

**SI Dataset S5 (554_SA_AUS_BA.csv)**

The .csv file containing site information for the 459 complete backed artifacts in the GMM analysis from southern Africa in addition to the 95 complete backed artifacts from Australia.

**SI Dataset S6 (SAfBA_environ.R)**

The R file containing the code used to complete the environmental analyses.

**SI Dataset S7 (SAfBA_environ.xlsx)**

The Excel file containing the data used in the environmental analyses.

**References**

1. K. S. Brown *et al.*, An early and enduring advanced technology originating 71,000 years ago in South Africa. *Nature* **491**, 590-593 (2012).

2. L. Wadley, Two'moments in time'during middle stone age occupations of Sibudu, South Africa. *Southern African Humanities* **24**, 79-97 (2012).

3. M. Will, N. J. Conard, Regional patterns of diachronic technological change in the Howiesons Poort of southern Africa. *PloS one* **15**, e0239195 (2020).

4. P. Villa, S. Soriano, N. Teyssandier, S. Wurz, The Howiesons Poort and MSA III at Klasies River main site, cave 1A. *Journal of Archaeological Science* **37**, 630-655 (2010).

5. W. Archer, C. M. Pop, P. Gunz, S. P. McPherron, What is Still Bay? Human biogeography and bifacial point variability. *Journal of Human Evolution* **97**, 58-72 (2016).

6. F. d’Errico *et al.*, Identifying early modern human ecological niche expansions and associated cultural dynamics in the South African Middle Stone Age. *Proceedings of the National Academy of Sciences* **114**, 7869-7876 (2017).

7. L. Wadley, Announcing a Still Bay industry at Sibudu Cave, South Africa. *Journal of Human Evolution* **52**, 681-689 (2007).
